# Distribution of Major and Trace Elements in a Tropical Hydroelectric Reservoir in Sarawak, Malaysia

**DOI:** 10.1155/2014/870187

**Published:** 2014-09-21

**Authors:** Siong Fong Sim, Teck Yee Ling, Lee Nyanti, Terri Zhuan Ean Lee, Nurul Aida Lu Mohd Irwan Lu, Tomy Bakeh

**Affiliations:** Faculty of Resource Science & Technology, Universiti Malaysia Sarawak, 94300 Kota Samarahan, Sarawak, Malaysia

## Abstract

This paper reports the metals content in water, sediment, macroalgae, aquatic plant, and fish of Batang Ai Hydroelectric Reservoir in Sarawak, Malaysia. The samples were acid digested and subjected to atomic absorption spectrometry analysis for Na, K, Mn, Cr, Ni, Zn, Mg, Fe, Sn, Al, Ca, As, Se, and Hg. The total Hg content was analysed on the mercury analyser. Results showed that metals in water, sediment, macroalgae, aquatic plant, and fish are distinguishable, with sediment and biota samples more susceptible to metal accumulation. The distributions of heavy metals in water specifically Se, Sn, and As could have associated with the input of fish feed, boating, and construction activities. The accumulation of heavy metals in sediment, macroalgae, and aquatic plant on the other hand might be largely influenced by the redox conditions in the aquatic environment. According to the contamination factor and the geoaccumulation index, sediment in Batang Ai Reservoir possesses low risk of contamination. The average metal contents in sediment and river water are consistently lower than the literature values reported and well below the limit of various guidelines. For fishes, trace element Hg was detected; however, the concentration was below the permissible level suggested by the Food and Agriculture Organization.

## 1. Introduction

Metals contamination has been a concern of hydroelectric development [1–5]. The process inevitably exposes rivers to the risk of metals contamination due to the alteration triggered in hydrological and sediment regime. The trace elements are often released into the aquatic environment from natural and/or anthropogenic sources where they are usually bound to sediment particles or soluble in water. These elements can then be taken up by aquatic organisms and transferred to human via food chain resulting in numerous adverse health effects; for example, methylmercury is a neurotoxin and exposure to arsenic increases the risk of skin cancer [6]. The bioaccumulation factor (BAF), expressed as the ratio of chemical concentrations in organisms over the concentrations in water [7], as high as 150−300 has been reported in fishes such as* Tilapia zilli*,* Tilapia guineensis*,* Clarias gariepinus,* and* Synodontis membranaceus* for various elements [8–12].

The construction of dam has been long challenged with the issue of elevated mercury (Hg). Upon impoundment of a dam, the naturally occurring inorganic Hg may be converted to bioavailable organic Hg by bacteria leading to bioaccumulation of Hg in fish [13]. The accumulation of Hg can be very persistent; for example, the methylmercury contamination reported in Canada and Finland took 20–30 years to be restored to the baseline level after impoundment [14]. Besides Hg, other elements were also reported to increase in sediment of Iron Gate, the largest dam and reservoir in Danube, 20 years after impoundment [15]. The potentials of metals contamination in dams and reservoirs have been revealed in numerous studies associating it with various anthropogenic inputs [16, 17].

Sarawak, a state in Malaysia on the island of Borneo, possesses high potential for hydroelectric development due to the abundant rainfall throughout the year [18]. A series of hydroelectric projects have been identified of which Batang Ai Hydroelectric Dam is the first dam impounded in 1985. The dam is a 29-year-old dam with the reservoir covering a total area of 9,000 ha and a capacity of 100 MW. The reservoir has been used for freshwater aquaculture activities where the production increases dramatically over years. In 1993, the production of tilapia fish was estimated at 22.9 metric tonnes (mt), but after 16 years, it increases by 13-fold to 298.9 mt and further soars to 488.8 mt in 2010 and 744.1 mt in 2011 [19].

According to Roulet et al. [20, 21], tropical soil is naturally rich in mercury which tends to be remobilized upon degradation of soil due to deforestation as well as flooding. The release of mercury into the environment due to the hydroelectric dam construction has been repeatedly reported where the element tends to be accumulated in aquatic organisms. The Hg level in fish of the affected system could take 15–30 years to be restored to its background level [22].

With the continuous increase of demand for aquaculture harvest and the potential of metal contamination particularly the phenomenon of mercury accumulation, the status of Batang Ai Reservoir is poorly understood. There is relatively little information on the metal accumulation in hydroelectric reservoir in this region; Barletta et al. [23] state that river basin and environmental management plans are poorly developed in tropical and subtropical countries. The mercury pollution policies are often not implemented leaving the risk of metal pollution undetermined. Thus, this paper attempts to evaluate the metal pollution status in Batang Ai Reservoir, 29 years after impoundment, reporting the major and trace elements in water, sediment, macroalgae, aquatic plant, and fish.

## 2. Materials and Methods

### 2.1. Sampling

**Figure 1:**
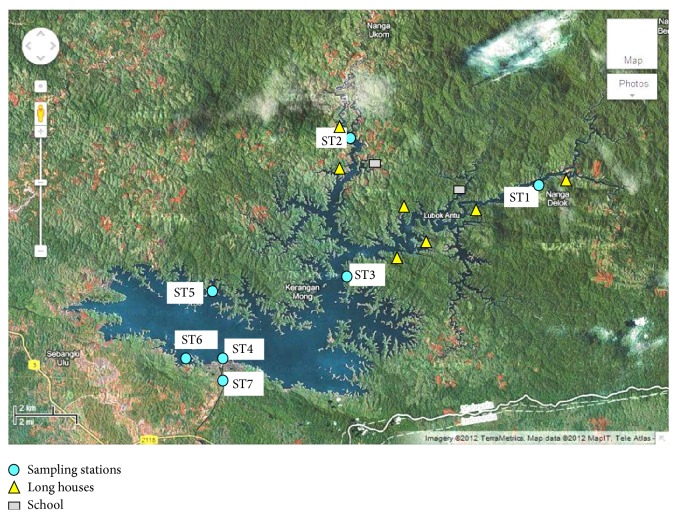
The sampling locations at Batang Ai Reservoir and the Ai River.

Water, sediment, macroalgae, aquatic plant, and fish samples were collected from 7 stations in Batang Ai Reservoir and the Ai River as shown in Figure 1. For water samples, a composite of triplicates was obtained from subsurface, acidified with 5 mL of 2 M HNO_3_, and stored at 4°C. Sediment samples were obtained using a grab sampler, stored in plastic bags, and kept at 4°C. For biological samples including fish, aquatic plant, and macroalgae, they were representative of the dominant species at respective stations in triplicate. Upon transportation of the samples to the laboratory, they were kept at −20°C until further analysis.  Table 1 summarises the species of plant and fish collected. Note that, in this paper, fish samples were only obtained from ST5 near the aquaculture site. Algae and aquatic plant were collected depending on the sample availability.

### 2.2. Acid Digestion and Metal Analysis

The samples were digested in triplicate and subjected to atomic absorption spectroscopy (AAS) for metal analyses including Na, K, Mn, Cr, Ni, Zn, Mg, Fe, Sn, Al, Ca, As, and Se. The total Hg was analysed on a mercury analyser (Perkin Elmer, FIMS 400). The water samples were analysed according to the standard method of APHA [27] where water samples were digested to free elements that are complexed. Samples were first filtered through 0.45 *μ*m membrane filter. Approximately 5 mL of concentrated HNO_3_ was added to 100 mL of water sample and put to slow boil on a hotplate to 10–20 mL, until the solution is clear. The sample was left to cool to room temperature and filtered through 0.45 *μ*m membrane filter. The filtrate was diluted to 100 mL for metal analysis.

For biological samples, they were washed under running tap water prior to drying to remove dirt. The dorsal of fish samples was dissected whilst for aquatic plant only the part above ground is considered. The samples were oven dried at 60°C and ground. A total of 0.5 g of sediment sample was digested with 6 mL of concentrated HNO_3_ and 2 mL of HCl on a hotplate until the solution is colorless. The sample was cooled and filtered through 0.45 *μ*m membrane filter and transferred to a 100 mL volumetric flask where the filtrate was diluted to the mark and mixed. For macroalgae and aquatic plant, 0.5 g of sample was digested in 6 mL of concentrated HNO_3_ whilst for fish 0.25 g of sample was digested in 6 mL of HNO_3_ and 1 mL of HCl [28]. All glassware was acid washed.

Detection limits of element analysed were Na (0.0037 ppm); K (0.0009 ppm); Mn (0.0016 ppm); Cr (0.0054 ppm); Ni (0.008 ppm); Zn (0.0033 ppm); Mg (0.0022 ppm); Fe (0.0043 ppm); Sn (0.21 ppm); Al (0.028 ppm); Ca (0.0037 ppm); As (0.12 ppm); Se (0.23 ppm); and Hg (at ppb level). Blanks were also analysed for potential contamination.

### 2.3. Statistical Analysis

The metal contents tabulated in tables with rows corresponding to samples and columns corresponding to variables (elements) were square rooted and standardised prior to principal component analysis (PCA). The multivariate exploratory approach reveals the clustering pattern of various samples and according to sampling locations. This facilitates the interpretation of a relatively large dataset whether metal contents in various samples are distinguishable and whether respective samples can be differentiated according to sampling locations. Pearson's correlation analysis was performed to identify the correlation between two elements where *P*value at 95% significance level was computed to evaluate the relationship.

### 2.4. Assessment of the Contamination Status

The contamination status is evaluated based on the contamination factor (CF), the geoaccumulation index (*I*_geo_), and the pollution index (PI). The contamination factor is expressed as the concentration of a given element in sediment, *C*_sample_, against the value of the average metal in the world surface rock, *C*_background_, stated by Martin and Meybeck [29]. The background levels of various metals are Mn (750 mg/kg), Cr (71 mg/kg), Ni (49 mg/kg), Zn (127 mg/kg), Mn (750 mg/kg), Hg (0.4 mg/kg), and Fe (35900 mg/kg). For CF < 1, the level of contamination is interpreted as low whilst 1 ≤ CF ≤ 3 is moderately contaminated, 3 ≤ CF ≤ 6 is contaminated, and CF > 6 is highly contaminated. Consider the following:

(1) $$\begin{array}{c} \text{CF}=\frac{C_{\text{sample}}}{C_{\text{background}}}. \end{array}$$

The geoaccumulation index, *I*_geo_, is calculated to illustrate the enrichment of metal concentration above the baseline concentrations [30]. According to Muller's classification, the sediment is classified as unpolluted if *I*_geo_ < 0, progressing from unpolluted to moderately polluted if *I*_geo_ is between 0 and 1, moderately polluted if *I*_geo_ is between 1 and 2, moving into polluted if *I*_geo_ is between 2 and 3, and polluted if *I*_geo_ is between 3 and 4. Consider the following:

(2) $$\begin{array}{c} I_{\text{geo}}=\log_{2}\left(\frac{C_{\text{sample}}}{{1.5*C}_{\text{background}}}\right). \end{array}$$

The pollution load index (PLI) is calculated as (CF_1_ × CF_2_ × CF_3_ × ⋯CF_*n*_)^1/*n*^, where *n* is the number of metals. The PLI value of >1 suggests the sediment is polluted whilst PLI < 0 implies unpolluted [31].

## 3. Results and Discussion

### 3.1. The Distribution of Heavy Metals in Water, Sediment, Aquatic Plant, Macroalgae, and Fish

**Figure 2:**
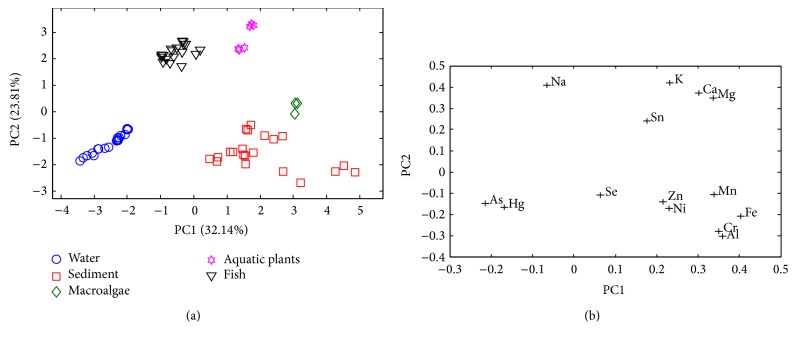
The scores plot of heavy metal contents according to heavy metals in water, sediment, aquatic plant, macroalgae, and fish.

Metals in water, sediment, macroalgae, aquatic plant, and fish are subjected to PCA yielding a scores plot of PC2 versus PC1 in Figure 2(a) with a total variance of 55.95%. The corresponding loadings plot is shown in Figure 2(b). Clearly, the distributions of metals in different samples are distinguishable where the loadings plot suggests that Hg and As are characteristic of water while Na is typical to fish.  Table 2 summarises the nonzero average of metal contents in respective samples. Essentially, elements such as Ca, Mg, and K are more prominent in sediment, macroalgae, aquatic plant, and fish with Na present in appreciable amount in fish. Aluminium on the other hand is profoundly detected in sediment due to the high content of silicoaluminate clays; nevertheless it is unlikely to be found in biological samples as Al in this form is insoluble. Under acidic condition, it may become more soluble and thus more available for plant uptake [32]. Other elements such as Fe, Mn, and Cr are variably detected in various samples, that is, sediment, macroalgae, aquatic plant, and fish, with Sn prominently present in macroalgae and plants. As a whole, metals tend to be found in higher concentrations in sediment corroborating its nature as metal bioaccumulators. The average trace metal contents specifically As, Cr, Ni, and Zn in sediment are relatively lower than those reported in a canyon reservoir in Southwest China [33]. They are also lower than those found in the hydroelectric dam of Danube [34]. In comparison to the selected metals, namely, Al, Zn, and Fe, identified in fishes of Tasik Mutiara, Malaysia, fishes at Batang Ai Reservoir are characterised by lower concentrations with As similarly undetected [35]. The level of Hg in fish on the other hand ranges between 0.03 and 0.20 mg/kg, comparatively lower than the range reported elsewhere, for example, in Tucuruí Reservoir and River Mojú (0.11–1.3 mg/kg) [36]; Newfoundland (0.13–0.86 mg/kg) [37]; Tanzania (0.003–0.263 mg/kg) [38]; Guizhou (0.3–0.5 mg/kg) [39]. Likewise Hg in sediment, at an average of 0.129 mg/kg, is less prominent than the level reported in two hydroelectric reservoirs in Quebec, Canada (0.15–0.49 mg/kg) [40]. Tin is found in elevated concentration in macroalgae and aquatic plants; according to Thompson et al. [41], the bioaccumulation factor of Sn in these samples can be as high as 100 where Nirbadha et al. [42] likewise revealed relatively large quantities of Sn in three aquatic plants in Kelaniya.

**Figure 3:**
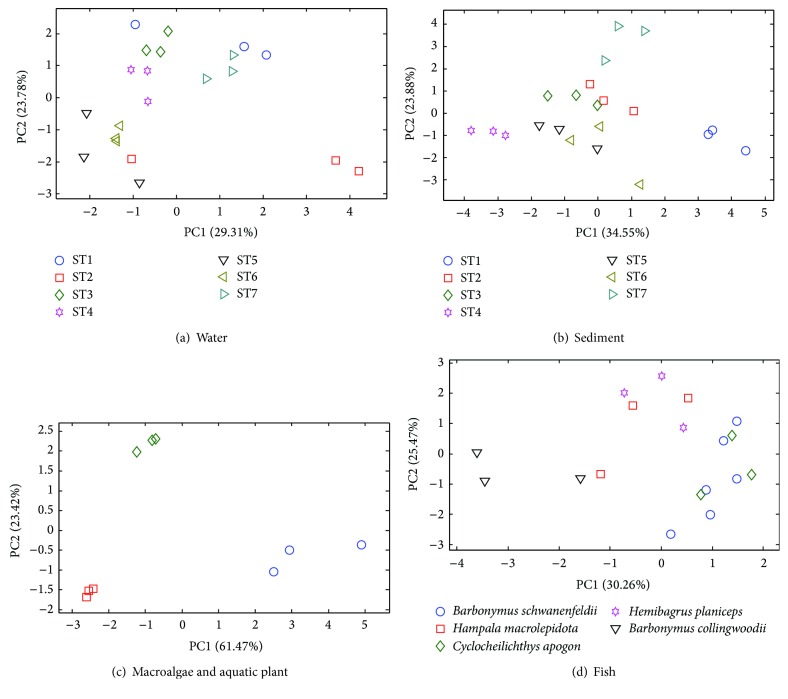
The scores plot of heavy metals in water, sediment, macroalgae, aquatic plant, and fish according to sampling stations.

**Figure 4:**
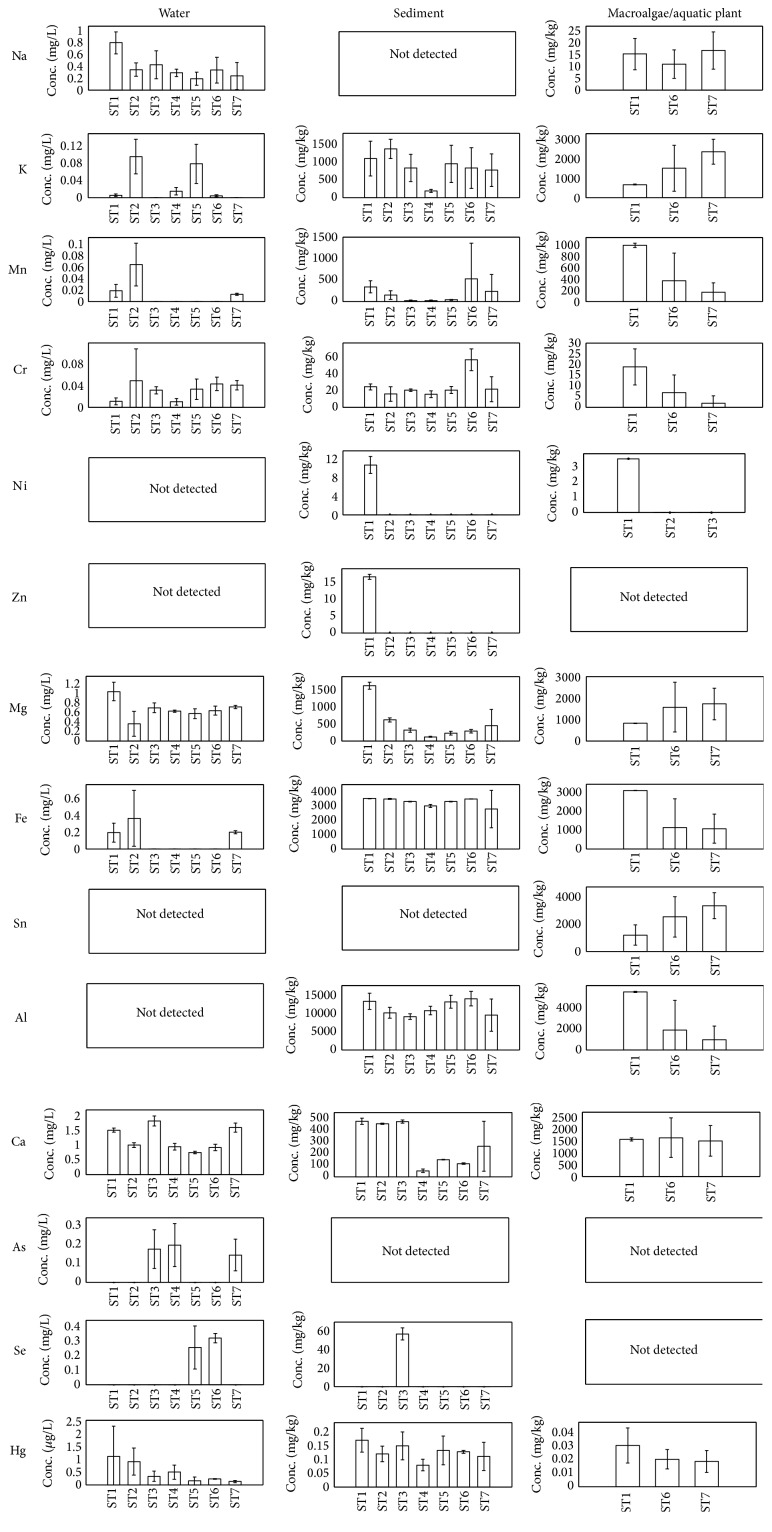
The average metal contents in water, sediment, macroalgae, and aquatic plant according to sampling stations.

The occurrence of metals in water, sediment, macroalgae, aquatic plant, and fish is examined independently with PCA; the scores plots are shown in Figure 3 according to sampling stations. For surface water, it appears that samples from the Ai River (ST1, ST3, ST4, and ST7) are primarily distributed over the upper region of the scores plot whilst samples from the aquaculture sites (ST5 and ST6 (abandoned)) are scattered around the lower right. The underlying pattern of metal distributions in sediment on the other hand suggests that samples from the upper stream (ST1), near the outflow (ST4), and downstream of the power house (ST7) are distinguishable. Macroalgae and aquatic plants collected from three different stations are seemingly differentiable. For fish, no distinctive pattern is interpretable.  Figure 4 illustrates the average metal concentrations in various samples according to sampling stations where the error bars indicate the standard deviations.

Evidently, Se is below the detection limit in most water samples except ST5 and ST6 with aquaculture activities. The presence of Se can be an indication of excessive discharge of fish feed as Se has been commonly added to animal feed including fish meal due to its importance in biological function [43, 44]. This element was consistently detected in fish feed by Alam et al. [45], Maule et al. [46], and Ikem and Egilla [44] at average concentrations of 5.4 mg/kg, 2.48 mg/kg, and 1.7 mg/kg, respectively. Ikem and Egilla [44] further examined the level of Se present correspondingly in aquaculture fish muscles with concentration ranges 0.2–0.4 mg/kg. As a whole, no significant correlation can be deduced between the waterborne and the accumulated Se in sediment. According to Canton and Derveer [47], the accumulation of Se in sediment does not correspond very well to the waterborne Se. It depends largely on the redox conditions where, under strongly reduced environment, insoluble selenium usually predominates [48]. As observed, Se is found in sediment at ST3 whilst the bioaccumulation of Se in aquatic plants is negligible.

Arsenic is detected in water at ST3, ST4, and ST7. This may be associated with the development and construction activity nearby as studies revealed that As is susceptible to leaching from construction debris with chromate-copper-arsenate (CCA) wood [49, 50]. At ST3, there are several long houses and a resort is located 4 km south of ST3, ST4 is near the outflow, and ST7 is downstream of the power house. Despite the presence of As in several stations, the element is undetected in other corresponding samples. Mn and Fe are found distinctively in water samples at ST1, ST2, and ST7. These elements are naturally present in soils; under reduced conditions, they tend to exist in soluble forms resulting in increased concentrations in water [51]. The distribution patterns of Mn and Fe in water are very similar with a correlation coefficient of 0.93 suggesting that both elements are geochemically correlated. Naturally, they are constituents of various source rocks, that is, igneous rock; in addition, they possess similar dissolution and precipitation behaviour under comparable redox conditions.

Sn, particularly organotin, has been widely used as a component in antifouling paint, applied as a finish coat to the submerged part of boat, and in pesticides. In this study, Sn is found in an appreciable amount in algae and plants. In fact, Sn is relatively immobile; it may exist as Sn(II) or Sn(IV) where both forms are readily precipitated under reduced conditions. The element can be profoundly accumulated in aquatic organisms. According to Thompson et al. [41], the bioaccumulation factors of tin in freshwater plant and fish could be as high as 100 and 3000, respectively. The present results likewise suggest that algae and aquatic plants are good indicators of Sn accumulation; its distribution is markedly higher near the aquaculture site (ST6) and the downstream of power station (ST7) possibly due to boating activities as well as the cooling system where tributyltin is commonly used in biocides. To date, there is no legislation in Malaysia to control the use of tributyltin. In this study, Cr is present at an average of 20 mg/kg in sediment of almost all stations except ST6 where higher concentration is also found in water. Despite the elevated concentration of Cr at ST6, there is no sign of bioaccumulation in the aquatic plants indicating that the element may not be bioavailable. For other elements such as Ni, Zn, and Mg, there is no concern of heavy metal contamination but interestingly they are detected distinctively in sediment at ST1 upstream.

**Figure 5:**
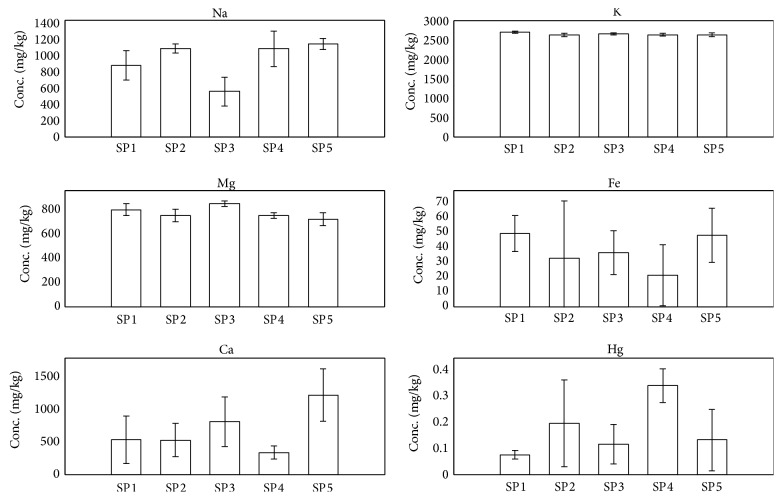
The average concentrations of six prominent elements in fish according to species and parts (SP1:* Barbonymus schwanenfeldii; *SP2:* Hampala macrolepidota; *SP3:* Cyclocheilichthys apogon; *SP4:* Hemibagrus planiceps; *SP5:* Barbonymus collingwoodii*).

Five species of fish samples were collected from the reservoir near the aquaculture farm at ST5. No well-defined separation can be interpreted based on the scores plot of PCA.  Figure 5 shows the average concentrations of six prominent elements according to species. Obviously, Hg seems to be bioaccumulated in higher concentration in* Hampala macrolepidota *and* Hemibagrus planiceps;* nevertheless the amount is generally well below the permissible level set by FAO at 0.5 mg/kg suggesting that the selected fishes are safe for consumption [52]. Other predominating elements are primarily major elements such as Na and K.

### 3.2. Correlation of Metals

The relationships of metals within samples, that is, water, sediment, and macroalgae/aquatic plant, are correlated based on the mean concentrations.  Table 3 summarises the results of Pearson's correlation analysis, where *P* < 0.05 are highlighted. The findings suggest that Mn and Fe in water are positively correlated whilst Na and Hg also demonstrate significant correlation. In sediment, on the other hand, positive correlations are statistically confirmed in Zn-Ni-Mg and Fe-Mn. For macroalgae/aquatic plants, Na-Mg are negatively correlated whilst Ca-Al correlate positively. The negative correlation, as suggested by Osaki et al. [53], indicates antagonistic mechanism in metal accumulation whereas positive correlation could imply that elements are taken via similar mechanisms. The correlations of metals between samples, that is, water-sediment, water-plant, and sediment-plant, are also evaluated. Apparently, no significant correlation is statistically deduced except Hg in water-plant (*r* = 0.99) and Ni in sediment-plant (*r* = 1).

Considering the metals uptake in algae and plant, macroalgae at ST1 demonstrates greater tendency of metal accumulation. According to Michalak and Chojnacka [54], macroalgae exhibits greater ability in accumulating heavy metals (more than 10 times) than vascular plants whilst studies elsewhere further support that* Enteromorpha* sp. is an excellent indicator for a wide range of metals including Fe, Al, Mn, Ni, and Zn [55–57]. The other plants species,* Polygonum* sp. and* Fimbristylis globulosa* at ST6 and ST7, respectively, are also possible metals accumulators; they have been employed for phytoremediation, treating water polluted with Cu, Cd, Zn, and Pb [58].

### 3.3. Assessment of Contamination Status

The contamination factors (CF) calculated for Mn, Cr, Ni, Zn, Mn, and Fe are comfortably below 1. The CF of Hg, however, is slightly above 0, at an average of 0.322, indicative of slight pollution. According to a survey of Hg present in 73 rivers of the USA, the concentrations reported range between 0.1 *μ*g/L and 5 *μ*g/L with two rivers exceeding 5 *μ*g/L (the limit for potable water supplies according to the Public Health Service) [59]. The unpolluted Hg level is defined at <0.1 *μ*g/L; with this criterion, the average Hg detected in the reservoir of 0.451 *μ*g/L is suspected to be affected by the anthropogenic inputs. Nonetheless, the concentration accumulated in the sediment, at an average of 0.129 mg/kg, is below the limit of 1 mg/kg set in sediment for attention [59]. The geoaccumulation indices, *I*_geo_, are less than 0 for all elements considered; according to Muller's classification, the sediment is categorised as unpolluted. The pollution load indices calculated across all stations (PLI) are consistently less than 1 suggesting no risk of contamination at the moment. The average metal concentrations in sediment and river water (as summarised in Table 1) are consistently lower than the literature values and are well below the limit set according to various guidelines such as the Food and Agriculture Organization Guidelines, 1985, Canadian water quality guidelines for the protection of aquatic life, interim freshwater sediment quality guidelines, and the probable effect level [24–26].

## 4. Conclusions

The findings of the study indicate that there is low risk of heavy metal contamination in the environment of Batang Ai Hydroelectric Reservoir. The aquaculture and development activities, however, may result in elevated Se, As, and Sn. The availability of the trace elements is largely governed by the redox conditions in the environment. Generally, there is no special concern of heavy metal contamination in fish; nonetheless there is a tendency of Hg bioaccumulation and continuous monitoring is necessary.

## Figures and Tables

**Table 1:** Summary of species of macroalgae, aquatic plant, and fish samples.

Fish (total length (cm))	Description	Algae/aquatic plants	Description
*Barbonymus schwanenfeldi*i (33.4 cm)	It is largely herbivorous, consuming aquatic macrophytes and submerged plants as well as algae.	*Enteromorpha* sp.	Found at ST1. It is commonly known as macroalgae.

*Hampala macrolepidota* (19.0 cm)	It is a migratory species where the diet consists mainly of aquatic insects.	*Polygonum* sp.	Found at ST6. It is a type of floating aquatic plant.

*Cyclocheilichthys apogon* (17.7 cm)	It is a carnivorous species consuming mainly small fish and aquatic insects.	*Fimbristylis globulosa *	Found at ST7. It is an emergent aquatic plant.

*Hemibagrus planiceps* (21.4 cm)	It is probably a predator feeding on crustaceans and smaller fishes.		

*Barbonymus collingwoodii* (18.6 cm)	It is largely herbivorous, consuming aquatic macrophytes and submerged plants as well as algae.		

**Table 2:** The nonzero average of metal contents in different samples.

Elements	Water (mg/L except Hg in *μ*g/L)	Sediment (mg/kg)	Macroalgae (mg/kg)	Aquatic plants (mg/kg)	Fish (mg/kg)	Literature range in sediment (mg/kg)	Literature range in water (mg/L and *μ*g/L if indicated with∗)
Na	0.396 ± 0.237	nd	15.32 ± 6.67	22.23 ± 2.71	949.45 ± 251.98	3190–6910^a^	—
K	0.053 ± 0.044	842.07 ± 4673.59	661.65 ± 24.02	2681.21 ± 142.23	2679.78 ± 42.78	7550–17300^a^	—
Mn	0.029 ± 0.022	273.86 ± 447.82	996.51 ± 37.44	72.95 ± 18.85	nd	348–5740^a^; 167–915^c^	4–86^a^; 0.038–0.239^c^; 0.2–10^d^; 0.05^e^
Cr	0.032 ± 0.023	24.55 ± 14.41	19.05 ± 8.54	nd	8.89 ± 2.17	40–343^a^; 30.2–74.8^b^; 92.5–160^c^; 37.3^f^; 90^g^	7-8^a^; 2.4–7.3^∗b^; 0.003–0.037^c^; 0.1^d^; 0.05^e^
Ni	nd	11.49 ± 1.96	nd	nd	nd	11.6–64.5^a^; 6.2–17.8^b^; <0.05^c^; 21^f^; 52^g^	9–18^a^; 1.2–5.4^∗b^; 0.005–0.035^c^; 0.2^d^
Zn	nd	16.48 ± 0.60	nd	nd	nd	54–904^a^; 21.8–127^b^; 182–484^c^; 123^f^; 315^g^	<5^a^; 26–88.3^∗b^; 0.016–0.066^c^; 2^d^; 5^e^
Mg	0.650 ± 0.216	527.69 ± 446.70	835.85 ± 11.12	1887.52 ± 837.31	775.87 ± 58.12	2400–7630^a^	11200–17300^a^
Fe	0.251 ± 0.179	3387.45 ± 207.37	3023.46 ± 8.13	966.79 ± 857.10	37.72 ± 18.25	8340–25400^a^; 12842–53581^c^	90–164^a^; 0.088–0.463^c^; 5^d^; 0.3^e^
Sn	nd	260.54 ± 63.68	1156.00 ± 721.40	3686.97 ± 448.67	nd		
Al	nd	10632.63 ± 2510.45	5292.01 ± 60.51	53.75 ± 44.18	31.51 ± 8.27	20000–52200^a^; 12280–35035^c^	20–98^a^; 0.024–0.149^c^; 5^d^
Ca	1.247 ± 0.403	321.79 ± 196.78	1487.69 ± 63.21	1498.24 ± 661.70	677.14 ± 422.52	16000–82100^a^	
As	0.168 ± 0.026	nd	nd	nd	nd	3.2–7.4^a^; 34.1–112.8b	11–14^a^; 13.1–47.7^∗b^
Se	0.289 ± 0.038	57.51 ± 5.49	nd	nd	nd		—
Hg	0.451 ± 0.503	0.1297 ± 0.042	0.03 ± 0.01	0.020 ± 0.001	0.158 ± 0.124	0.17–0.35b	0.01–0.06^∗b^

^a^Blackfoot River watershed [24].

^b^Port Klang coastal area [25].

^c^Nile Delta [26].

^d^Food and Agriculture Organization Guidelines, 1985 [26].

^e^Canadian water quality guidelines for the protection of aquatic life [26].

^f^Interim freshwater sediment quality guidelines [26].

^g^Probable effect level [26].

^*^indicates unit in *μ*g.

**Table:** (a) Water

	Na	K	Mn	Cr	Mg	Fe	Ca	As	Se	Hg
Na	1.00									
K	−0.34	1.00								
Mn	0.15	0.61	1.00							
Cr	−0.51	0.39	0.40	1.00						
Mg	0.75	−0.71	−0.42	−0.65	1.00					
Fe	0.25	0.40	**0.93**	0.32	−0.16	1.00				
Ca	0.46	−0.61	−0.07	−0.12	0.55	0.14	1.00			
As	−0.24	−0.51	−0.40	−0.32	0.05	−0.33	0.46	1.00		
Se	−0.35	0.15	−0.39	0.34	−0.18	−0.50	−0.62	−0.54	1.00	
Hg	**0.78**	0.19	0.64	−0.36	0.25	0.62	0.10	−0.34	−0.50	1.00

**Table:** (b) Sediment

	K	Mn	Cr	Ni	Zn	Mg	Fe	Al	Ca	Se	Hg
K	1.00										
Mn	0.25	1.00									
Cr	0.02	−**0.84**∗	1.00								
Ni	0.28	0.35	−0.02	1.00							
Zn	0.28	0.35	−0.02	1.00	1.00						
Mg	0.51	0.39	−0.06	−**0.95**∗	−**0.95**∗	1.00					
Fe	0.64	0.34	0.35	0.39	0.39	0.43	1.00				
Al	0.13	0.58	0.62	0.41	0.41	0.28	0.55	1.00			
Ca	0.70	−0.04	−0.32	0.46	0.46	0.64	0.39	−0.34	1.00		
Se	−0.03	−0.39	−0.14	−0.17	−0.17	−0.18	0.05	−0.51	0.45	1.00	
Hg	0.64	0.29	0.17	0.66	0.66	0.68	0.63	0.32	0.68	0.34	1.00

**Table:** (c) Aquatic plants and macroalgae

	Na	K	Cr	Mg	Fe	Al	Ca	Hg
Na	1.00							
K	−0.47	1.00						
Cr	0.14	0.59	1.00					
Mg	−**0.93**∗	0.61	0.20	1.00				
Fe	−0.29	0.68	0.10	0.18	1.00			
Al	0.43	−0.38	−0.20	−0.62	0.24	1.00		
Ca	0.13	−0.23	0.07	−0.21	0.17	−**0.83**∗	1.00	
Hg	0.43	−0.70	−0.44	−0.46	−0.79	−0.11	−0.41	1.00

^*^ *P* < 0.05.
